# Supplementary material for: Are carbon emissions trading and green financial instruments synergistic? -Comprehensive quantitative research based on content analysis
Source: PLoS One. 2024 Mar 7;19(3):e0298601. doi: 10.1371/journal.pone.0298601 (PMC10919601; doi:10.1371/journal.pone.0298601)
Supplement: S1 Data — (ZIP) [file pone.0298601.s001.zip › Data/地级市Z_F工作报告.zip!/╣≤╓▌/┴∙┼╠╦«╩╨/└┤╫╘╣1⁄2╓┌║┼í╛┬φ┐╦╩2╛▌═°í┐_2008.docx]

各位代表：

　　现在，我代表市人民政府，向大会作工作报告，请予审议，并请各位政协委员和列席的同志提出意见。

　　一、2007年政府工作回顾

　　过去一年，我们高举中国特色社会主义伟大旗帜，坚持以邓小平理论、“三个代表”重要思想为指导，深入贯彻落实科学发展观，紧紧依靠全市各族干部群众，开拓进取，扎实工作，排难而进，全市经济社会发展呈现出稳定增长、效益提高、活力增强、民生改善的良好态势。

　　（一）经济发展较快，运行质量和效益较好。全市生产总值完成300.55亿元，增长16%。全社会固定资产投资完成170.22亿元，增长16.7%。财政总收入和地方财政收入分别完成46.08亿元和21.61亿元，分别增长23.97%和28.2%。规模以上工业增加值完成114.7亿元，增长19.9%。全市金融机构存、贷款余额分别完成270.38亿元和206.63亿元，增长18.9%和21.9%。社会消费品零售总额完成77.59亿元，增长16.5%。城镇居民人均可支配收入为11125元，增长20.9%。农民人均纯收入为2348元，增长17.2%。

　　（二）“三农”工作进一步加强，农业农村面貌发生可喜变化。支农惠农政策全面落实，全市共兑现涉农补助1.55亿元。粮食产量达82.16万吨，增长2.7%，再创历史新高。农业结构调整加快，种养业结构趋于合理，规模化水平逐步提高。马铃薯、蔬菜种植面积不断扩大，分别达126万亩和22万亩；畜牧业产值占农业总产值的比重达42.2%。农业产业化经营步伐加快，产业化经营组织已达176个，共带动13.6万农户实现增收2.62亿元。农业农村基础设施得到改善，完成中低产田土改造2.62万亩和坡改梯工程1万亩，解决了9.57万人和2.1万头牲畜的饮水困难，建成通乡油路124公里和通村公路1508公里，建成沼气池12400口，完成8000户农村贫困户住房改造。农民培训力度加大，共培训 34.36万人次，转移农村剩余劳动力7.3万人。农产品加工发展加快，农产品商品率不断提高。城市、企业、民间的资金、技术、人才正向农村转移，以城带乡、以工促农的良好局面逐步形成。

　　（三）产业结构调整初见成效，发展势头向好。传统支柱产业做大做强，产业链延长，产品附加值提高，产业门类和产品种类增多，发展方式逐步从粗放向集约转变。大企业在结构调整中的支撑作用明显。盘江煤电（集团）公司原煤产量突破千万吨大关，响水、松河、发耳等大型骨干矿井建设加快，地方煤矿通过资源整合，平均单井生产能力已达7.2万吨/年，全市原煤产量完成4397万吨；煤炭产业链正向煤化工延伸，老鹰山煤化工项目开工建设，盘县红果恒鼎和盘东北循环经济型煤焦化项目即将动工，盘南煤化工项目前期工作加快。全省单机容量和总装机容量最大的盘南发电厂建成投产，一批电源项目加速推进，全年新增电力装机120万千瓦，完成发电量195.43亿度，增长13.2%。水城钢铁（集团）公司钢产量迈上300万吨台阶，年销售收入突破100亿元，进入全省“百亿企业”行列。总日产1万吨熟料新型干法水泥生产线陆续开工建设；利用粉煤灰、煤矸石等工业废弃物发展新型建材的步伐加快。矿山机械长足发展，综采液压支架、掘进机、重型刮板运输机等整机装备制造企业相继建成。有色金属工业加快发展，并向后端加工延伸，六盘水双牌铝业公司形成了年产20万吨电解铝、8万吨铝板的生产能力；电解锌已形成一定规模。轻工业、食品加工业等加快发展。第三产业增加值增长17.1%，占生产总值的比重达34.3%。全年实现旅游总收入9.88亿元，增长49.46%。商贸流通、住宿餐饮等服务业正朝着标准更高、质量更好的方向发展。

　　（四）基础设施建设取得新进展，发展条件不断改善。镇胜高速公路六盘水境内段建成通车，水盘高速公路开工建设，红威、俄发、英柏、柏火等在建二级公路达180多公里；六沾铁路复线进场施工；六盘水月照机场预可研报告已按程序上报待批。完成3座病险水库除险加固工程，建成“三小”水利工程1万个，新增、恢复和改善有效灌溉面积8.26万亩，治理水土流失面积44.2平方公里。全年新增通信光缆1375公里。电子政务和政府门户网站建设跻身全国中小城市先进行列。全市邮政所有的农村自办网点完成了电子化改造。“十件实事”完成较好。

　　（五）节能减排成效显著，生态建设扎实推进。广泛深入地开展了节能减排宣传，强化了节能环保等市场准入标准，落后生产力淘汰力度加大，加强了重点领域、重点企业的监控，形成了党政重视、企业重视、全民重视的良好工作格局。全市企业共投入环保资金8.65亿元，较上年增加一倍。41家重点监控的用能企业单位产品能耗指标稳定降低率为65％。全市万元生产总值能耗预计较上年下降5.5%，二氧化硫和化学需氧量排放总量分别下降9.98％和5.64％，省下达我市的节能减排任务全面完成。以退耕还林、天然林保护、珠防林等为重点的林业生态建设力度加大，完成营造林面积14.77万亩,实施市级石漠化治理试点2万亩，建成市中心城区环城林带700余亩，全市森林覆盖率已达35.1%。

　　（六）城市建设力度加大，城市形象进一步提升。《六盘水城市总体规划（2006－2020）》已经省人民政府批准实施。城市基础设施建设力度加大，凉都大道东段建成通车、人民路中段改造基本完成，51条小街小巷综合改造成效明显；水城河综合治理加快推进；10万立方米城市燃气储气柜扩建工程投入使用；凉都体育中心、市人民医院综合住院大楼开工建设。“整脏治乱”深入开展，城市管理不断加强。红果、平寨城市基础设施和功能进一步完善，一批重点小城镇建设步伐加快。

　　（七）重点领域改革继续深化，对外开放不断扩大。国有企业改革稳步推进，完成了17户市属国有企业清产核资的集中联审认定工作，省属国有企业分离移交学校工作有序进行。财政体制改革进展顺利，国库集中支付、部门预算和政府采购等全面实施。启动了农村综合改革试点工作，六枝特区集体林权制度改革试点全面完成。金融体制改革步伐加快，六盘水市商业银行获准组建。分配制度改革有序开展，机关事业单位工资制度改革基本完成。非公有制经济加快发展，实现增加值91.89亿元，增长 25.2%。招商引资取得较好成效，实际到位资金49.79亿元，增长18.47%。

　　（八）社会事业加快发展，民生问题逐步解决。各级财政用于教育、卫生、就业、社会保障等方面的支出达23.46亿元，增长32.36%。教育基础设施不断改善，建成3所农村寄宿制学校，完成农村薄弱学校改造40所，市中心城区6所中小学基本建成；教育“两基”成果得到巩固，以县为主的农村义务教育经费保障机制基本建立，落实农村义务教育阶段“两免一补”政策资金5866.16万元；普通高中教育规模继续扩大；中等职业教育完成招生1.2万人，增长20%；六盘水理工学院申办工作进展顺利。新型农村合作医疗覆盖面继续扩大，参合率达87.42%，共为农民报销医药费6484.23万元。就业再就业工作超额完成全年目标，新增城镇就业2.1万人，帮助1342户“零就业家庭”至少1人实现就业，城镇登记失业率为4.3%。基本养老、基本医疗、失业、工伤等保险扩面征缴任务超额完成，生育保险启动实施。两次提高城市居民最低生活保障标准，月人均较上年增加50元，7.34万城镇低收入人口受益。农村低保制度全面实施，23.86万农村贫困人口基本生活得到保障。全年救助受灾群众25.47万人次。农村贫困人口比上年净减少9200人。全市用于农村贫困家庭和城镇低保家庭的民用煤补助达8000万元。实行了廉租住房租赁补贴，建成经济适用住房7.37万平方米。国有重点煤矿采煤沉陷区综合治理工程全面推进。通过积极发展生产、切实加强市场监管、发放困难群体物价补贴等，缓解了物价上涨带来的压力。人口计划生育工作水平稳步提高，人口自然增长率为5.73‰，符合政策生育率达95.5%。文化、体育、广播电视等社会事业继续发展，成功举办了第四届凉都消夏文化节，完成了216个20户以上自然村广播电视“村村通”工程。 安全生产形势总体平稳，其中煤炭百万吨死亡率为2.16，继续处于全省先进水平。

　　过去一年，我们不断加强民主法制建设和政府自身建设。自觉接受人大的法律监督、工作监督和政协的民主监督。村（居）民委员会换届全面完成。政务公开、厂务公开、村务公开和部门办事公开不断扩大。“五五”普法深入开展，依法治市全面推进。社会治安、来信来访、应急管理等进一步加强。“平安六盘水”创建工程、禁毒工作等取得重大进展。荣获“全国双拥模范城”称号。监察、审计工作力度加大，廉政建设得到加强。机关作风教育整顿活动成效明显，行政效能不断提高。科技、民族、宗教、气象、人防、档案、地方志、对台、外事侨务等取得新成绩，工会、妇女、儿童、老龄、残疾人、红十字等事业继续发展。

　　各位代表！以上成绩的取得，是省委、省政府坚强领导，省直各部门大力支持的结果；是市委正确领导，市人大、市政协监督支持的结果；是全市各族人民团结奋斗，社会各界关心支持的结果。在此，我代表市人民政府，向全市工人、农民、知识分子、干部，向各民主党派、工商联、无党派人士、人民团体，向驻市人民解放军指战员、武警部队官兵、公安政法干警，向所有关心、支持我市发展的各界人士，表示衷心的感谢并致以崇高的敬意！

　　在肯定成绩的同时，我们也清醒地看到，经济社会发展中还存在不少困难和问题。主要是：农民稳定增收的基础不牢，城乡居民收入差距继续拉大；经济发展对能源资源的依赖程度较高，资源环境保护、生态建设、节能降耗等压力较大；融资渠道不宽，投资主体单一，社会投资不足；煤、电、油、运等要素性约束问题突出，经济调节难度加大；上学、看病、就业、物价等涉及群众切身利益的不少问题还没有得到很好解决；人口计生、安全生产等工作基础依然薄弱；维护社会稳定工作与群众要求还有一定差距；政府自身建设亟待进一步加强，发展环境特别是软环境与形势发展的要求还不适应。对于这些问题，我们将采取措施，认真加以解决，努力把政府工作做得更好。

　　二、2008年政府工作的目标和要求

　　今年是全面贯彻落实党的十七大精神的第一年，是实施“十一五”规划承上启下的关键一年。政府工作的总体要求是：高举中国特色社会主义伟大旗帜，以邓小平理论和“三个代表”重要思想为指导，深入贯彻落实科学发展观，全面贯彻落实党的十七大精神，紧紧围绕转变经济发展方式，继续解放思想，坚持改革开放，抢抓发展机遇，坚持好字优先、能快则快，加快工业化、推进城镇化、带动农业产业化，切实加强农业基础建设，着力调整产业结构，大力推进节能减排和生态环境建设，更加注重改善民生和促进社会和谐，奋力开创六盘水市又好又快发展的新局面。

　　国民经济和社会发展的主要预期目标是：生产总值增长16%以上；全社会固定资产投资增长15%以上；财政总收入增长20%以上，其中地方财政收入增长18%以上；全社会消费品零售总额增长16%以上；城镇居民人均可支配收入增长8%以上，农民人均纯收入增长8%以上；居民消费价格总水平涨幅控制在4.5%以内；城镇登记失业率控制在4.2%以内；人口自然增长率控制在7‰以内；单位生产总值能耗下降4.71%，主要污染物减排2%。

　　做好今年的政府工作，正确处理“好”与“快”的关系至关重要。要始终坚持好字优先，全面贯彻落实科学发展观，更加注重结构调整，更加注重创新驱动，更加注重统筹兼顾，更加注重民生问题，努力实现发展质量效益速度相协调、投资消费出口相协调、人口资源环境相协调、改革发展稳定相协调。要在确保好字优先的前提下，立足我市发展实际，充分发挥我市比较优势，努力做到能快则快。第一，六盘水市经济总量小、人均水平低的基本市情，决定了必须快。如果没有一个较快的发展速度，就业、教育、社会保障、医疗卫生等许多民生问题就难以解决，与发达地区的差距就难以缩小，在资源配置、争取投资和扩大市场方面面临的竞争压力就难以缓解。第二，六盘水市在区域经济发展中和省的盘子里所处的战略地位和肩负的责任，决定了需要快。随着区域位置及资源供给半径优势进一步凸显，省内外对我市能源原材料产品的需求十分旺盛，要求我们在能源原材料供应上作出更大的贡献。第三，六盘水市已经具备了许多有利条件，决定了能够快。经过多年的建设发展，基础条件不断改善。特别是“十五”期间经济社会发展的快速积累，去年各项目标任务的全面超额完成，为做好今年的工作奠定了坚实基础。国家继续实施西部大开发战略，加大对西部地区的转移支付力度，为我们争取支持提供了可靠的政策机遇；省委、省政府提出毕水兴经济带和大城市发展战略，为我市加快发展创造了良好条件。今年我市重大项目多、投入大、涉及面广，项目的实施，将对我市长远发展产生深远影响，对当前发展产生有力拉动。全市上下在贯彻落实科学发展观中，加快发展的意识进一步增强，谋发展的积极性提高，推进发展的措施更加有力，形成了干事创业的浓厚氛围。对此，我们一定要紧紧扭住发展这个第一要务，聚精会神搞建设，一心一意谋发展，只要是有质量、有效益的投入，能搞多大搞多大；只要是有质量、有效益的速度，能搞多快搞多快。

　　今年1月中旬以来，我市遭受了强度大、持续时间长达1个多月的雪凝天气灾害，工农业生产损失严重，群众生活受到较大影响。面对严重的自然灾害，在党中央、国务院的关怀下，在省委、省政府的领导下，在社会各界的大力支持下，各级干部以身作则、靠前指挥，各有关部门和单位迅速行动、全力以赴，人民解放军、武警部队和公安民警勇挑重担、顽强拼搏，全市上下万众一心、众志成城，千方百计保交通、保供应、保民生，夺取了抗灾救灾的阶段性胜利，展示了六盘水人特别能战斗、特别能吃苦、特别能奉献的精神。事实证明，只要紧紧依靠广大人民群众，充分发挥社会主义制度能够集中力量办大事的政治优势，就一定能够战胜前进道路上的各种挑战和困难。我们一定要大力弘扬这种精神，把灾后恢复重建工作和经济社会发展紧密结合起来，切实做好生产自救和灾后重建工作，切实安排好受灾群众生活，全力夺回灾害造成的损失，努力实现全年经济社会发展目标。

　　三、促进全市经济又好又快发展

　　今年，我们将着力抓好以下八个方面的工作。

　　（一）加强“三农”工作。坚持把解决好“三农”问题作为全部工作的重中之重，认真落实各项强农惠农政策措施，加快建立和完善以工促农、以城带乡的长效机制，进一步加大“三农”投入，不断加强农业基础建设，积极发展现代农业，大力发展乡镇企业，多渠道增加农民就业，促进农民增收，扎实推进社会主义新农村建设，加快改变农业农村面貌。

　　稳定发展粮食生产。全面落实各项扶持政策措施，保护和调动农民的种粮积极性，切实稳定粮食播种面积；大力实施粮食增产工程，着力扩大良种良法普及率，努力提高粮食单产，确保粮食增产2%以上。

　　大力调整农业结构。加大种养业结构调整力度，使种养业结构渐趋合理，重点加快发展马铃薯、蔬菜、畜牧养殖“三大产业”,逐步实现区域化布局、规模化生产、专业化经营。马铃薯产业要继续抓好品种繁育、科技推广、市场销售“三大体系”建设。全面完成150万亩种植面积，建好6万亩高产示范基地。大力推进与省科技厅、省农科院开展的马铃薯产业发展科技合作。蔬菜产业要按照集中连片的要求，加快市、县两级蔬菜基地建设，新建商品蔬菜基地3万亩，重点建好郎岱片区、百车河片区商品蔬菜基地。因地制宜发展反季节蔬菜、无公害蔬菜，积极引导农民扩大蔬菜种植面积，在提高本地蔬菜市场供给率的同时，加快走出去步伐。畜牧业要以推广种草养畜为突破口，促进畜牧业大发展大提高，力争畜牧业产值占农业总产值的比重增加1个百分点以上。在加快发展生猪养殖的同时，抓好以牛羊为重点的畜禽品种改良，继续实施好盘县和水城县草地生态畜牧业科技扶贫养羊项目，建好盘县优质肉羊基地、水城县优质肉牛基地和钟山区优质肉鸡基地；加强动物疫病防治，确保畜牧业健康发展。

　　加快发展农业产业化经营。围绕提高农产品商品率，积极培育壮大龙头企业，促进生产、加工、储运和销售一体化，加快形成公司、基地与农户之间合理的利益联结机制。规范发展农业专业合作社和农村经济服务组织，充分发挥涉农公司在农业产业化经营中的作用，提高农民进入市场的组织化程度。整合农村信息资源,进一步办好农经网。建好农村“万村千乡市场工程”，积极改善农产品流通条件。

　　加强农业和农村基础设施建设。完成中低产田土改造3.5万亩。建成“三小”水利工程1万个，大力实施烟水配套工程，新增、恢复和改善有效灌溉面积4.5万亩，治理水土流失面积40平方公里。继续推进农村电网改造，加快实现城乡同网同价。积极发展农村洁净能源，建成沼气池1.1万口。加快推进以村寨道路硬化、改厕改圈、排水排污、绿化美化为主要内容的村寨建设，不断改善农村人居环境。

　　强化农业科技推广和农民素质培训。加强基层农技推广人才队伍建设，加快农业科技研发和先进适用技术推广应用，推动农业科技成果进村入户。整合培训资源，提高培训质量，完成“绿色证书”培训6000人、“阳光工程” 培训1万人、“雨露计划” 培训4000人、农民工就业培训1万人。完成250名村卫生员、200名村兽医员招生任务。充分发挥现代远程教育培训农民的作用。以县为主不断加强村干部培训，着力提高村干部素质。 深入推进新阶段扶贫开发。整合扶贫资源，捆绑使用各类扶贫资金。切实把扶贫工作重点放在促进农村低收入人口增收上来，扎实抓好整村推进、产业扶贫、连片开发，逐户落实增收措施，有针对性地开展扶贫，减少农村低收入人口3.83万人。继续坚持领导干部联系点和机关单位定点扶贫制度，鼓励工矿企业就近自愿结对帮扶，动员各方面力量参与农村扶贫开发。

　　（二）着力优化工业结构。充分发挥我市在能源原材料产业上的比较优势，按照走新型工业化道路的要求，调整优化工业结构，推动经济增长由主要依靠增加物质资源消耗向主要依靠科技进步、劳动者素质提高、管理创新转变，进一步发展壮大工业经济，加快把我市发展成为现代化工业城市。当前的主要任务是，改造提升传统支柱产业，并在此基础上加快发展新材料产业，同时大力发展装备制造业和轻工业，促进产业产品结构优化合理。

　　继续做大做强煤炭、电力、钢铁、建材等传统支柱产业。大力推进地方煤矿资源整合，加快建设响水、发耳、格目底、松河等骨干矿井，争取开工建设新华、化乐等煤矿；加快发耳电厂建设，力争动工建设六枝电厂和四格风力发电项目一期工程，有序推进响水、万家口子、毛家河、膳泥坡等水电项目建设；加快实施水城钢铁（集团）公司铁及铁前系统、中厚板生产线等技术改造工程，推进首都钢铁公司进入我市发展“煤（焦、化）－钢－电”一体化项目前期工作；加快建设总日产1万吨熟料新型干法水泥生产线，大力推进六盘水恒远建材公司年产45万立方米粉煤灰砌块、野马寨电厂粉煤灰制蒸氧砖及混凝土砌块等新型建材项目。

　　加快延长传统产业链，发展煤化工、新型铝等新材料产业，推进精深加工，提高产品附加值。大力推进老鹰山煤化工项目，开工建设盘县红果恒鼎和盘东北循环经济型煤焦化项目，扎实做好盘南煤化工项目前期工作，继续开展保田－青山煤层气资源风险勘探。加快发展有色金属工业，重点推进六盘水双牌铝业公司年产电解铝35万吨、铝板加工15万吨技改扩能工程。

　　大力发展矿山机械、机电产品，逐步把装备制造业发展成为支柱产业。重点支持水城矿业（集团）公司机械制造分公司、六盘水煤机厂、六枝工矿（集团）公司宏狮公司等企业，加快采煤机、综掘机、综采液压支架、单体液压支柱、金属铰接顶梁、皮带刮板输送机、矿车、防爆电机车等产品、设备的开发、生产步伐。

　　积极发展轻工业、食品加工业、农产品加工业。加大对六盘水啤酒公司等企业的扶持力度，支持市食品总厂等马铃薯加工企业加快发展，积极为面粉、皮革等加工业做大规模创造条件。

　　推进工业结构调整，要切实做好以下工作。第一，科学合理规划工业布局，用规划指导产业结构调整，引导产业向特定区域集中，促进主体功能区的形成。第二，加快建设工业集中区，推进集群化发展，促进产业聚集和上下游产业耦合。今年，要争取红山石桥工业集中区的建设取得实质性进展，尽快达到企业入驻条件。积极规划建设六枝特区和盘县的工业集中区。第三，进一步推进地企共建，强化大企业在结构调整中的主体地位，支持企业开展技术创新，推动产业链纵向延伸；鼓励企业之间进行强强联合，实现优势互补和多元化发展，推动产业链横向整合。第四，加强经济运行调度。进一步完善工业经济预警预测及分析制度，抓好能源、运输、资金等统筹协调，实现工业经济正常平稳运行。

　　（三）努力扩大固定资产投资。一是大力拓宽资金来源和投资渠道。加强与上级的沟通和联系，积极争取国家和省加大对我市的投入。加大招商引资力度，引进知名企业、战略投资者到我市投资兴业。充分发挥大企业、大项目在扩大投资中的主渠道作用，积极推进跨区域、跨行业组建大型企业集团，支持以项目为载体、以资产为纽带，以产权多元化和投资主体多元化扩大投资规模。积极发展资本市场,加快金融生态环境建设，争取金融机构加大对我市的支持力度；鼓励有条件的企业上市融资；探索民间资金进入资本市场的有效途径。二是进一步优化投资结构。在国家宏观调控政策的框架内，把扩大投资的着力点放在基础设施、能源原材料、生态环境保护、社会事业、民生问题等重点领域和薄弱环节上，放在调整结构、转变发展方式、实施可持续发展上，坚决防止盲目投资和低水平重复建设。三是加强项目建设和管理。建立重大项目领导干部包保联系制度，扎实推进项目前期工作，切实为项目建设搞好服务。围绕加速项目落地，着力办好土地、环评等关键性手续。坚持质量第一，提高建设质量。优化施工组织，大力整治建设项目周边环境，加快建设进度。

　　（四）加强基础设施建设。继续加大以交通、电网、水利、信息为重点的基础设施建设力度，加快改善我市经济社会发展条件，特别是要集中力量尽快完成基础设施的灾后恢复重建工作。加强以公路为重点的交通基础设施建设，力争公路建设质量实现大的提高；大力推进水盘高速公路建设，建成红威、英柏、俄发等二级公路，力争开工建设六枝经郎岱至毛口二级公路，新建通乡油路150公里和通村公路1200公里，深入推进县、乡公路养护管理体制改革；切实做好矿区运煤公路的规划、建设和管护；抓紧编制老鹰山经六枝至大山哨高速公路的规划；配合做好六沾铁路复线建设、火车站换边和货场搬迁，力争六盘水月照机场立项，开工建设毛口码头。围绕工业布局、城市发展，完善电网规划，加快电网建设，着力推进500千伏变电站前期工作，建成220千伏盘县红果变、北郊变二期扩建工程和110千伏六枝片区木岗、盘县淤泥等重点输变电工程，尽快缓解供电基础设施的瓶颈制约。加快推进农村饮水安全工程，解决农村10万人饮水安全问题；在完成10座病险水库除险加固的基础上，力争再争取实施10座病险水库的除险加固工程；大力推进市中心城区防洪二期工程；争取动工建设白河沟水利工程，积极推进双桥、岱翁等供水工程的前期工作。以现有的网络为基础，加大资源整合的力度，加快电子政务统一网络平台建设，深化电子政务应用，完成市级电子政务二期工程建设；进一步提高电话、互联网入户普及率；加快数字电视网信息基础设施建设，积极推进互联网、通讯网、电视网“三网融合”，提高信息覆盖广度和深度。

　　（五）加快城镇化进程。启动实施《六盘水城市总体规划（2006－2020）》。进一步强化市中心城区作为市域中心城市的地位，加快大城市建设步伐；按照建设区域性中心城市的要求，加快发展红果、平寨两个市域次中心城市；有重点地发展主要交通沿线的小城镇，加大市中心城区周边卫星城镇建设力度，对工矿区有序实施城镇化改造。围绕提高承载力，加大城市基础设施建设投入，加快完善城镇道路、水、电、气、绿化、通讯和污水、垃圾处理等基础设施。围绕扩大城市规模，按照“适度超前、因地制宜、突出特色”的要求，抓紧编制城市路网规划，以路网建设构建大城市框架，合理布局城市空间；优化城市功能，提高城市综合服务能力。围绕提高城市竞争力，强化经营城市的理念，继续打造“凉都”城市品牌，提高对外知名度，实现城市自身增值；对城市土地、基础设施、公共物品等国有资产资源，通过重组、拍卖、租赁、转让、抵押、有偿使用、冠名等多种经营运作方式，盘活存量资产，促进城市财富的节约与增长。围绕提高城镇化水平，加快推进城镇户籍、住房、教育、就业、社会保障等制度改革，促进农村人口向城镇有序转移。围绕做大城市经济，大力发展第三产业，加快改变经济增长过分依赖资源型产业的格局，重点发展旅游业、交通运输业、邮电通讯业、物资供销仓储业、金融保险业、信息服务业、房地产业、新闻出版业、市政公用事业、社区服务业、文化卫生体育服务业、住宿餐饮服务业，提高服务业在三次产业中的比重，尽快使服务业成为我市经济的主导产业。

　　按照建设综合承载力强、环境优美、文明有序的现代化城市目标，进一步加大市中心城区的建设和管理力度。一是继续加强城市道路建设。在抓好东西向城市主干道建设的基础上，加快南北向主干道建设；全面建成凉都大道，开工建设人民路东、西段和凤凰路东延伸段、碧云路东延伸段，扩建城南路（钟山大道与凉都大道连接段），打通龙井路（人民路与钟山路连接段），加快钢城大道北段建设步伐，继续实施小街小巷综合改造。二是大力推进“绿色城市”建设。实施以山体、城市绿地为重点的城市绿化工程；加快市中心城区环城林带建设；继续实施水城河综合治理，在确保水变清的同时，对沿河两岸有序进行公园化改造。三是加强城市管理和城市环境综合整治。切实把城市管理的重心下移到社区，推进社区的标准化建设、规范化管理、制度化运作，提高城市管理水平；继续深入开展“整脏治乱”；进一步规范公交车和出租车经营秩序，大力整治车容车貌。

　　（六）加强节能减排和资源环境保护。坚持把节能减排、环境保护和节约资源作为转变经济发展方式的重要抓手，不断增强可持续发展能力。

　　全力打好节能减排攻坚战、持久战。加快淘汰高耗能、高排放、低效益的落后生产能力。严格执行国家产业政策和行业准入标准，从源头上防止高耗能、高污染、低效益项目进入。以电力、冶金、建材等高耗能行业和年耗能5000吨标准煤以上企业的节能降耗工作为重点，着力抓好重点领域、重点行业和重点企业的节能降耗监控。强化企业的主体责任和社会责任，鼓励企业利用新技术、新工艺、新设备实施节能减排重大技术改造，努力实现能耗稳定下降和污染物稳定达标排放。认真执行国家节能减排统计、监测、考核实施方案和办法，落实节能减排工作责任制。力争开工建设红果、平寨生活污水处理工程。加强循环经济建设，争取实施盘县发电厂以大代小项目，积极推广盘江煤电（集团）公司煤泥干化利用、水城矿业（集团）公司瓦斯发电、水城钢铁（集团）公司节气减污等循环发展模式，加快推进大宗工业废弃物的规模化综合利用和产业化发展。大力推广新材料、新技术，抓好建筑、交通运输等行业节能降耗工作。加大宣传力度，深入开展节能减排全民行动，努力形成以政府为主导、企业为主体、全社会共同推进的工作格局。

　　加强环境保护和生态建设。严格实行环境影响评价、容量总量控制、“三同时”和排污许可证等制度。做好污染源普查工作。加大农村面污染源治理力度。加强饮用水源地环境保护。强化环境执法，严厉查处违法行为。完善环境和地质灾害监测预警体系与环境监察体系，严格执行地质灾害环境恢复治理保证金管理制度,加强资源开发过程中的地质灾害防治和生态环境保护。加快实施国有重点煤矿采煤沉陷区综合治理工程。认真落实国家关于完善退耕还林的政策措施，巩固退耕还林成果。启动4个县（特区、区）国家石漠化综合治理试点工程。继续抓好天然林保护、珠江防护林等重点林业生态建设工程，完成11万亩营造林任务。

　　合理利用和保护资源。大力促进节约用地和集约用地，强化土地利用总体规划的整体控制作用，在加强耕地特别是基本农田保护的同时，努力保障建设用地需求；积极开展对闲置土地和工矿废弃地的开发利用，推进土地有偿使用制度改革，盘活存量土地，提高土地利用效率和集约化程度。加大土地开发整理力度，努力实现耕地占补平衡。积极推进矿产资源有偿使用制度改革，继续整顿和规范矿产资源开发秩序，推广先进的开采工艺和技术，提高资源回收率。合理配置水资源，统筹协调生活、生产和生态用水。加强野生动植物资源保护。切实做好有害生物检疫、防治工作。

　　（七）积极推进改革开放。坚持把深化改革作为加快发展的根本动力，扎实抓好重点领域改革。以发展为取向，以“三改革一加强”为重点，稳步推进国有企业改革，继续抓好市县国有企业改革，积极配合省属驻市企业做好破产关闭、分离企业办社会职能等工作。切实抓好农村各项改革，稳定和完善土地承包关系，依法规范土地经营权流转，大力推进以“三改革一化解”为重点的农村综合改革，全面实施集体林权制度改革。加快财政金融体制改革，继续深化部门预算、国库集中支付和政府采购等改革，进一步加强收支两条线管理。挂牌成立六盘水市商业银行，大力支持农村信用社改革与发展。

　　坚持把扩大开放作为促进发展的重要途径，大力改善投资软环境，加快走出去、引进来的步伐，促进人才、资金、技术、管理等生产力要素流向我市。改进招商引资方式，把招商与选商、引资与引智结合起来，突出针对性和实效性，努力提高招商引资的质量和水平。积极参与东部沿海发达地区的区域合作，进一步加强与周边地区、对口帮扶城市和友好城市的协作。

　　（八）促进非公有制经济加快发展。毫不动摇地鼓励、支持、引导非公有制经济加快发展。加大政策支持的力度，坚持非禁即允，破除阻碍非公有制经济发展的观念和体制障碍，推进公平准入，在投资核准、财税政策、土地使用等方面，与其他所有制企业享受同等待遇。加强对非公有制企业的项目引导、技术支持、制度建设、管理创新，提高企业可持续发展能力和核心竞争力。大力实施“中小企业成长工程”，加快完善信用担保和再担保体系，拓宽中小企业融资渠道。加强对非公有制企业的监管，促进其依法经营，引导其积极承担社会责任。

　　四、加强以改善民生为重点的社会建设

　　解决好民生问题，是政府最大的公务。要把更多的精力用在解决民生问题上，用在为人民群众创造舒适、优美的生活环境上，用在促进人的全面发展上，努力使全体人民学有所教、劳有所得、病有所医、老有所养、住有所居，提高人民的幸福水平。

　　（一）优先发展教育事业。切实做好迎接国家“两基”教育督导检查的各项工作，进一步巩固“两基”成果，推进义务教育均衡发展；建立教育资助制度，保障经济困难家庭、进城务工子女平等接受教育，促进教育公平。认真落实国家实施城乡免费义务教育政策，完善义务教育经费保障机制，启动农村义务教育历史债务化解工作，建成“农村义务教育阶段薄弱学校改造工程”项目学校20所。大力发展普通高中教育，着力提高教育质量和初中毕业生接受普通高中教育的比重。加快发展以中等职业教育为重点的职业教育。继续办好六盘水职业技术学院。加强六盘水师范高等专科学校软硬件建设，力争六盘水理工学院申办成功。统筹抓好学前教育、民族教育、特殊教育、远程教育，支持和规范民办教育。加强教师队伍建设，努力解决教师缺额问题，大力开展农村中小学教师“回炉培训”。切实重视素质教育。加强人才培养和使用，以大专院校、大企业、大项目为载体，积极引进各类高层次人才、急需人才。强化各类科技人才队伍建设，加大科普力度，切实发挥科学技术在发展中的推动作用。

　　（二）加快发展卫生事业。坚持公共医疗卫生的公益性质，加强公共卫生体系建设，强化医德医风教育，提高医务人员素质，努力为群众提供安全、有效、方便、价廉的医疗卫生服务。进一步完善农村三级卫生服务网络，完成200个甲级村卫生室建设。大力推进城市社区卫生服务体系建设，新建8个城市社区卫生服务机构。加快市人民医院综合住院大楼建设步伐，抓好水钢总医院的搬迁建设工作，积极推进惠民济困医院建设。全面启动实施城镇居民基本医疗保险。认真落实新型农村合作医疗新的补助标准，不断扩大新型农村合作医疗覆盖面，力争参合率达90%以上。深入开展爱国卫生运动。加强人口计生工作，打牢基层基础，继续做好流动人口的管理和服务，综合治理出生人口性别比失衡问题，稳定低生育水平，确保人口出生率控制在12.8‰以内，符合政策生育率达95.5%以上。

　　（三）大力发展文化等社会事业。坚持社会主义先进文化前进方向，推动文化大发展大繁荣。倡导和弘扬“三线”文化，挖掘和保护民族民间文化，打造和发展品牌文化和特色文化。多渠道加大文化基础设施建设投入，加快构建公共文化服务体系，启动建设947个行政村文化信息资源共享工程，建成六盘水乌蒙山国家地质公园博物馆，完成199个20户以上自然村广播电视“村村通”工程，实施好广播电视无线覆盖工程。继续整理文化典籍，认真做好地方志工作。办好建市30周年庆典活动和凉都消夏文化节。积极推进文化体制改革。加强文化市场监管，着力整治网吧经营秩序。广泛开展全民健身运动，积极发展竞技体育。加快凉都体育中心建设。

　　（四）切实做好就业再就业和社会保障工作。实施扩大就业的发展战略，促进以创业带动就业，多渠道开发就业岗位，实现城镇新增就业1.9万人、下岗失业人员再就业1800人。积极开展就业援助，着力解决“零就业家庭”、失地农民的就业困难。建立企业职工工资正常增长机制和支付保障机制，落实并逐步提高最低工资标准。规范和协调劳动关系，全面落实国家对农民工的政策，依法维护劳动者合法权益。继续扩大城镇职工基本养老、基本医疗、失业、工伤、生育等保险的覆盖面，确保参保人数分别达4.86万人、23.6万人、8.3万人、14.75万人和4.13万人。继续实施好城乡最低生活保障制度，动态地做到应保尽保。大力推进城乡社会帮扶救助体系建设，加强防灾减灾救灾工作。积极发展社会福利和慈善事业。做好老龄工作，加快养老服务机构建设，建成20所农村敬老院。

　　（五）加快完善住房保障体系。完成8000户农村贫困户住房改造。继续实施廉租住房制度，对县城以上符合住房困难条件规定、申请廉租住房租赁补贴的低保家庭基本做到应保尽保。加快经济适用住房建设，逐步改善城镇低收入家庭住房困难。规范房地产开发，加大住房供应结构调整力度，重点发展中低价位、中小套型普通商品住房，增加住房有效供应。积极推进工矿棚户区改造。

　　（六）努力保持物价基本稳定。高度重视城乡居民的“菜篮子”和“米袋子”问题，大力发展生猪、蔬菜等农副产品生产，加强重要物资储备体系建设，畅通“绿色通道”，增加市场供应，保障居民基本生活必需品不断档、不脱销，尤其要加强粮食市场调控，完善粮食储备体系，保障粮食安全。以事关民生的商品价格为重点，加强物价监测、预警和调控，对部分重要商品及服务实行临时价格干预措施，适时开征价格调节基金。加强市场监管，严查严惩搭车涨价、串通涨价、哄抬物价、扰乱市场、欺行霸市、行业垄断等违法行为。

　　（七）全力维护社会安定和谐。牢固树立“以人为本，安全发展”的理念，正确处理安全与生产的关系，切实做到不安全不生产，抓安全促生产。坚持“安全第一、预防为主、综合治理”的方针，强化领导责任、主体责任、监管责任、岗位责任，以煤矿、交通为重点,大力开展安全隐患排查治理，坚决遏制重特大安全事故发生，推动安全生产形势持续稳定好转。切实加强应急管理工作，推进应急体系建设，加快形成“政府统筹协调、社会广泛参与、防范严密到位、处置快捷高效”的应急管理工作机制，不断提高预防和应对突发事件能力。继续开展产品质量安全专项整治，保障人民群众健康安全。深入推进“平安六盘水”建设，加快建立健全以群防群治为基础的社会治安防控体系，严厉打击“两抢一盗”等违法犯罪活动。巩固禁毒成果，继续打好禁毒人民战争。切实做好人民群众来信来访工作，认真解决土地征用、房屋拆迁、企业改制等方面群众反映的突出问题，保障人民群众的合法权益。加强库区移民搬迁安置工作，落实后期扶持政策措施，维护库区稳定与和谐。兼顾地方政府、矿山企业、矿区群众“三者”利益，建设“和谐矿区”。

　　（八）加强社会主义精神文明和民主法制建设。深入开展群众性精神文明创建活动，加强社会公德、职业道德、家庭美德、个人品德建设，倡导和谐理念，培育和谐精神，提高公民道德水准，形成文明风尚。自觉接受人大及其常委会的法律监督和政协的民主监督，高度重视民主党派、工商联和无党派人士的意见、建议，支持工会、共青团和妇联等人民团体更好地发挥作用，接受新闻舆论和社会公众监督。巩固和扩大基层民主，推进村（居）民自治。深入开展“五五”普法教育。加快少数民族地区经济社会发展，维护民族团结。加强国防教育，继续推进“双拥”共建。大力实施《六盘水市妇女儿童发展规划纲要（2000－2010）》，发展妇女和青少年事业。关心残疾人的就业和生活。继续做好宗教、外事、侨务、对台、档案、人防、气象等各方面工作。

　　五、加强政府自身建设

　　按照“务实、高效、公正、清廉”的要求，进一步加强政府自身建设，营造又好又快发展的政务环境。

　　（一）继续解放思想，建设创新政府。加强公务员教育和培训。适应新形势需要，积极更新知识，不断提高推动科学发展的思想水平和业务素质。以改革开放暨建市30周年庆典活动为契机，继续解放思想，进一步振奋精神，增强开拓创新能力，打破束缚，破解难题，不断创新工作方法，丰富工作内容，完善工作机制，打开工作局面，创造性地落实中央和省的决策部署。

　　（二）加快转变职能，建设服务政府。坚持市场出效率、政府保公平，进一步转变政府职能，在加强和改善经济调节、市场监管的同时，把更多的精力和资源用在加强社会管理上，用在提高公共服务水平上，全方位、全过程地为大企业发展和大项目建设搞好服务、为非公有制经济及外来投资者搞好服务、为人民群众搞好服务。认真贯彻执行《政府信息公开条例》，继续推进政务、厂务、村务公开和公用部门办事公开，拓宽人民依法直接行使民主权利的渠道，保障公民的知情权、参与权、表达权、监督权。

　　（三）严格依法行政，建设法治政府。深入贯彻落实行政许可法和全面推进依法行政实施纲要，严格按照法定权限和程序履行职责。规范行政执法，加强执法监督，强化执法责任，全面提高政府依法行政能力。大力提倡公道正派、公平公正，严肃整治政府工作人员行政不作为和乱作为问题，优化经济发展环境。

　　（四）创新行政管理，建设效能政府。深化行政管理体制改革，进一步理顺部门职责关系，健全部门之间的协调配合机制，努力解决机构重叠、职能交叉、权责脱节、政出多门的问题，提高行政效率。坚持简政放权、分级负责。推进事业单位分类改革。大力推进行政服务中心和行政投诉中心建设，继续精简和规范行政审批。坚持科学民主决策，完善重大问题集体决策制度、专家咨询、社会公示和听证、决策责任追究等制度。加强行政效能监察和政务督查，建立行政首长问责制，推动工作落实。

　　（五）树立优良政风，建设务实政府。坚持“一手抓适应，一手抓提高”，既要解决最紧迫、最现实的问题，又要多干打基础、利长远的事情，努力创造经得起实践、历史和群众检验的业绩。大力精简会议、文件和应酬，腾出时间和精力想大事、议大事、抓大事，深入实际、深入基层、深入群众开展调查研究。扩大政府公共服务的覆盖范围，加大对社会事业和民生的投入，有计划、有步骤地解决涉及群众切身利益的热点难点问题，认真办好2008年“十件实事”。

　　（六）强化廉洁从政，建设廉洁政府。坚持综合治理、标本兼治、惩防并举、注重预防的方针，进一步加强廉政建设。建立健全决策权、执行权、监督权既相互制约又相互协调的权力结构和运行机制，拓展从源头上防治腐败的工作领域，旗帜鲜明地反对腐败。切实加强审计、监察等政府内部监督，严防失职渎职、违法违纪现象发生。牢记“两个务必”，强化行政成本效益观念，坚持勤俭办一切事业，建设节约型机关。

　　各位代表！完成今年的目标任务，实现对人民群众的庄严承诺，使命崇高，责任重大。我们一定要紧密地团结在以胡锦涛同志为总书记的党中央周围，全面贯彻落实党的十七大精神，继续解放思想，开拓进取，大力推动科学发展，着力构建和谐社会，奋力开创六盘水市改革发展稳定的新局面，为谱写六盘水人民美好生活的新篇章而努力奋斗！
